# Endovascular coiling versus surgical clipping for the treatment of unruptured cerebral aneurysms: Direct comparison of procedure-related complications

**DOI:** 10.1097/MD.0000000000019654

**Published:** 2020-03-27

**Authors:** Xiao-kui Kang, Sheng-fu Guo, Yi Lei, Wei Wei, Hui-xin Liu, Li-li Huang, Qun-long Jiang

**Affiliations:** a Department of Neurosurgery, Liaocheng People's Hospital, Liaocheng, Shandong; b Department of Gynaecology and Obstetrics, Anyi County People's Hospital, Nanchang; c Department of Neurology, Mianyang Central Hospital, Sichuan; d Department of Medical Examination; e Department of Endocrinology, Liaocheng People's Hospital, Liaocheng, Shandong, China.

**Keywords:** endovascular coiling, surgical clipping, unruptured cerebral aneurysms

## Abstract

**Background:**

Endovascular coiling and surgical clipping are routinely used to treat unruptured cerebral aneurysms (UCAs). However, the evidence to support the efficacy of these approaches is limited. We aimed to analyze the efficacy of endovascular coiling compared with surgical clipping in patients with UCAs.

**Method:**

A systematic search of 4 databases was conducted to identify comparative articles involving endovascular coiling and surgical clipping in patients with UCAs. We conducted a meta-analysis using the random-effects model when I^2^> 50%. Otherwise, a meta-analysis using the fixed-effects model was performed.

**Results:**

Our results showed that endovascular coiling was associated with a shorter length of stay (WMD: −4.14, 95% CI: (−5.75, −2.531), *P* < .001) and a lower incidence of short-term complications compared with surgical clipping (OR: 0.518; 95% CI (0.433, 0.621); *P* < .001), which seems to be a result of ischemia complications (OR: 0.423; 95% CI (0.317, 0.564); *P* < .001). However, surgical clipping showed a higher rate of complete occlusion after surgery, in both short-term (OR: 0.179, 95% CI (0.064, 0.499), *P* = .001) and 1-year follow-ups (OR: 0.307, 95% CI (0.146, 0.646), *P* = .002), and a lower rate of short-term retreatment (OR: 0.307, 95% CI (0.146, 0.646), *P* = .002). Meanwhile, there was no significant difference in postoperative death, bleeding, and modified Rankin Scale (mRS) > 2 between the 2 groups.

**Conclusions:**

The latest evidence illustrates that surgical clipping resulted in lower retreatment rates and was associated with a higher incidence of complete occlusion, while endovascular coiling was associated with shorter LOS and a lower rate of complications, especially ischemia.

## 1 Introduction

Subarachnoid hemorrhage (SAH) accounts for approximately 5% of all stroke cases, and in addition to rapidly increasing morbidity rapidly, an increasing trend in the youth rate is obvious.^[1]^ Based on clinical experience, the most common precipitating factor for SAH is ruptured cerebral aneurysms, with morbidity of 9 per 100 000 people.^[2]^ Although a few researchers have reported a stroke rate of approximately 2% per year, which indicates a decline over the past 2 decades, the rate of SAH decrease was smaller than that of stroke.^[2]^ Furthermore, the prevalence of unruptured cerebral aneurysms (UCAs) ranges from 1.7% to 3.1% in the aggregate population;^[3]^ UCAs lead to rupture or death within 1 month for 50% of patients, and incapacitation appears in 40% of patients who survive more than 1 month.^[4,5]^ Therefore, UCAs require timely attention, by using traditional craniotomy aneurysm clipping and interventional embolization, to prevent them from rupturing. Due to its microinvasive nature, the use of endovascular coiling is increasing at a rapid pace,^[6,7]^ and several investigations have found that the clinical results of endovascular coiling are superior to those of clipping for treating UCAs.^[8,9]^ Endovascular coiling avoids a craniotomy and a large incision, which shortens healing time and reduces the incidence of perioperative complications.^[10–12]^ Zhang et al^[13]^ deemed that the hospital stay following interventional therapy was much shorter than that for surgical clipping. However, the intensive promotion of emerging implantable devices, such as coils and stents, might lead to an excessive financial burden and procedure-related complications.^[13]^ On the other hand, a large number of studies have demonstrated that surgical clipping is associated with better durability in aneurysmal obliteration, even though clipping is more invasive.^[10,11,14]^ In this regard, we conducted a meta-analysis to compare the safety and efficiency between endovascular coiling and surgical clipping in patients with UCAs.

## 2 Materials and methods

Our review work was conducted in accordance with the Preferred Reporting Items for Systematic Reviews and Meta-Analyses (PRISMA) guidelines.^[15]^

### 2.1 Data sources and searches

The search for comparative articles involving endovascular coiling and surgical clipping in patients with UCAs was conducted by 2 authors (Q-LJ and X-KK) using electronic databases including Cochrane Library, Medline (1966–2019.7), PubMed (1966–2019.7), and EMBASE (1980–2019.7). The MeSH search terms were as follows: “unruptured aneurysms, endovascular coiling, surgical clipping.” Meanwhile, we manually checked the reference lists of the retrieved articles to search for other potential qualifying trials until no more articles could be identified.

### 2.2 Inclusion and exclusion criteria

Identified studies from the literature search were then further evaluated for inclusion. Inclusion criteria were as follows: population: participants with UCAs; intervention: included endovascular coiling and surgical clipping for treating UCAs; comparison: results about the treatment-related complications are provided; outcome measures: 1 of 10 endpoints length of stay (LOS), postoperative bleeding, death, complications, ischemia, vasospasm, hydrocephalus, completed occlusion, modified Rankin Scale (mRS) > 2, and retreatment were accessed); and full-text studies officially published in English.

Studies were excluded if they evaluated the outcomes between endovascular coiling and surgical clipping without reporting our specified study outcomes in the 2 groups; they were study designs other than clinical studies, such as case reports/series (< 10 patients), reviews, letters to editor, and meta-analyses; they studied animals instead of humans; or the data was unbalanced between the patient populations.

### 2.3 Data extraction and endpoints

We assigned 2 reviewers (X-KK and Q-LJ) to independently complete this part. A third author joined the extraction process in cases of disagreement. Basic demographic information (age, sex, diagnosis, number of included cases, and outcomes) was extracted. The primary study endpoint measurements were those relevant to surgical safety (postoperative bleeding, death, and short-term complications especially for ischemia, vasospasm, and hydrocephalus). The secondary endpoint was a composite of complete occlusion and postoperative mRS > 2, which were associated with procedural efficiency. Other outcomes of interest were the composite of LOS and retreatment. The clinical outcomes that appeared in the hospital or within 30 days were defined as short-term outcomes.

### 2.4 Statistical analysis

Stata version 11.0 was applied for the statistical analyses. The heterogeneity was assessed with the help of the I^2^ statistic. I^2^ values lower than 50% were regarded as indicative of low heterogeneity, and a fixed-effects model was used. Otherwise, a random-effects model was applied. For dichotomous outcomes (postoperative bleeding, death, complications, ischemia, vasospasm, hydrocephalus, completed occlusion, mRS > 2, and retreatment), the odds ratios (ORs) or rate differences (RDs) with 95% confidence intervals (CIs) were used for analysis. Alternatively, the weighted mean difference (WMD) combined with the 95% CI was used for continuous outcomes (LOS).

## 3 Results

### 3.1 Search results

**Figure 1:**
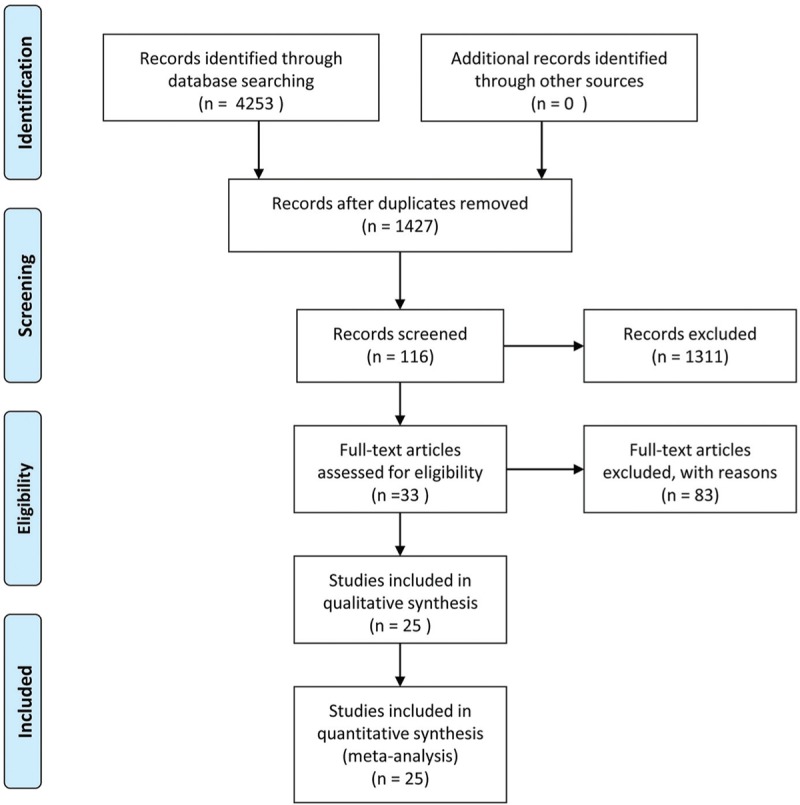
Flowchart of the study selection process.

A total of 4253 studies were identified by searching the electronic databases; there were 2826 articles after removing duplicates, and 1311 articles were excluded according to our preplanned eligibility criteria by reviewing the title and full abstract. There were 83 studies excluded after scanning the full text. Finally, 25 articles were eligible for quantitative synthesis. Figure 1 describes more information about the searching process.

### 3.2 Quality assessment and study characteristics

The Newcastle-Ottawa Scale (NOS) was used to evaluate the quality of the included non-RCT (randomized controlled trial). One RCT was regarded as high quality with a Jadad score of 5. The quality scores for all articles are shown in Table 1. A total of 25 studies,^[16–40]^ including 129,317 participants, were included in our meta-analysis. Of these 129,317 patients with UCAs, 72,010 were assigned to the endovascular coiling group, and 57,307 were assigned to the surgical clipping group; the sample size for each included study varied from 41 to 64,043. The studies were from the USA, Germany, Netherlands, Canada, China, Japan, Norway, Korea, France, and Switzerland. Table 2 describes the study characteristics in more detail.

**Table 1:**
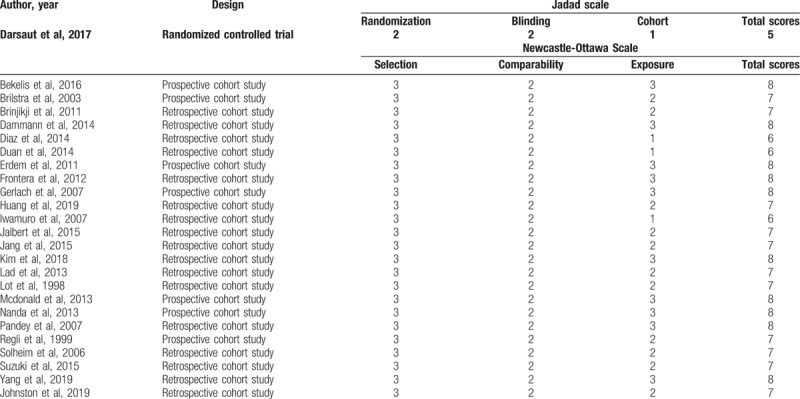
Quality assessment scores of the included studies.

**Table 2:**
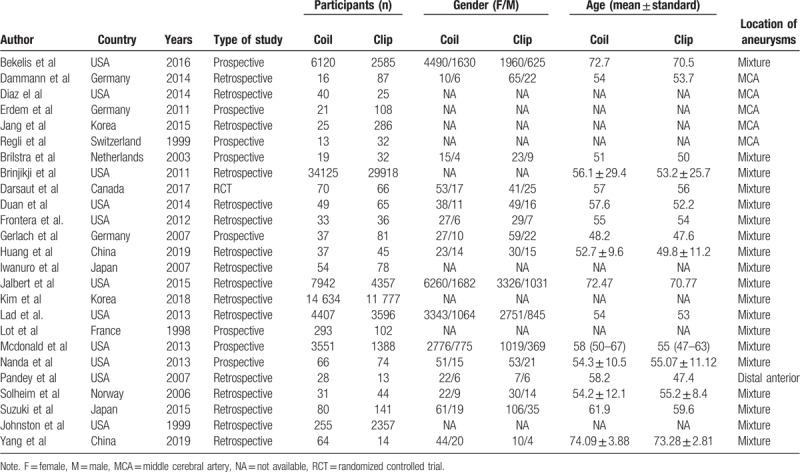
Overview of included studies.

### 3.3 Primary endpoints

**Figure 2:**
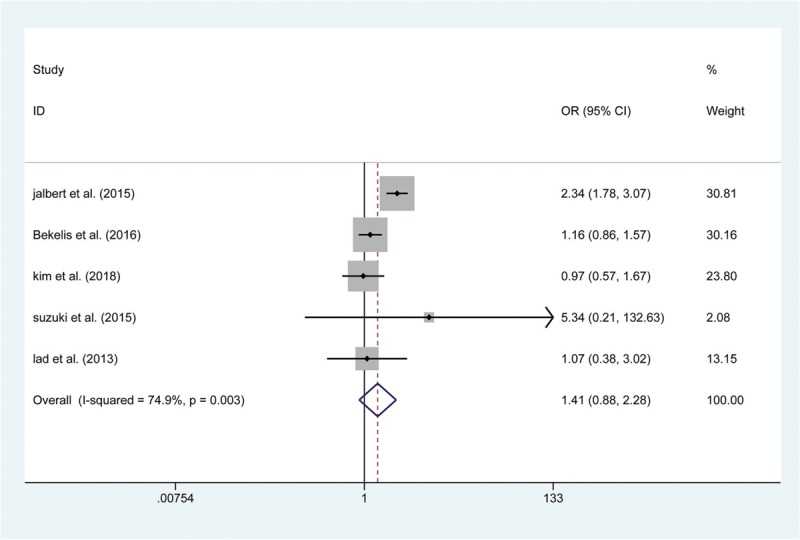
Forest plot of odds ratio (OR) of death within 30 days with endovascular coiling versus surgical clipping.

**Figure 3:**
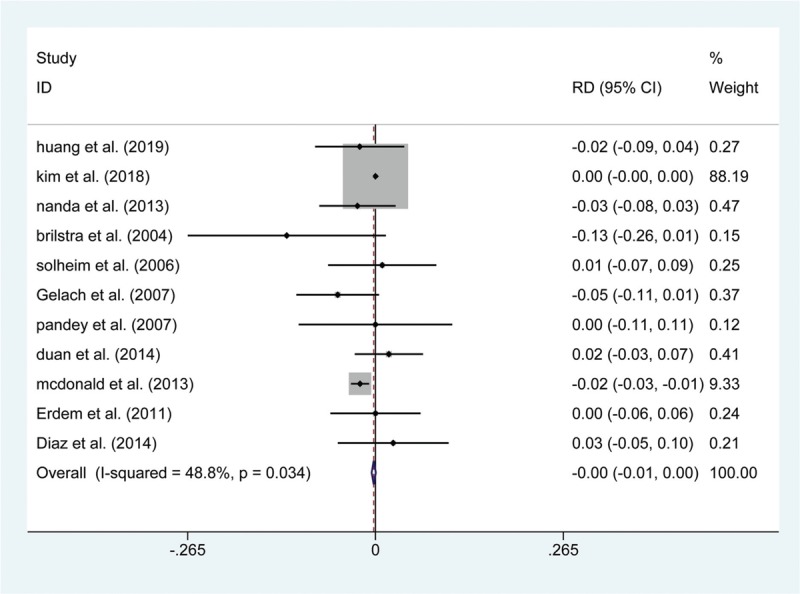
Forest plot of rate difference (RD) of periprocedural bleeding at short-term follow with endovascular coiling versus surgical clipping.

**Figure 4:**
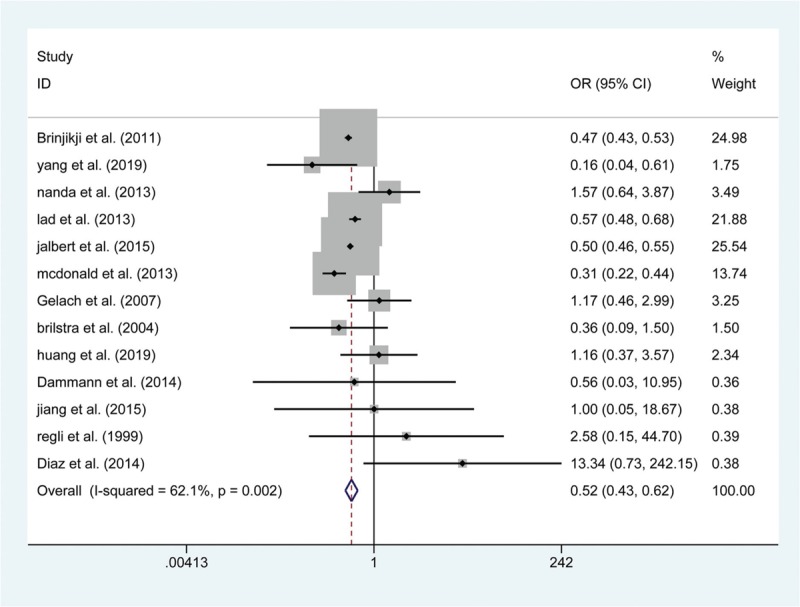
Forest plot of odds ratio (OR) of postoperative complication at short-term follow with endovascular coiling versus surgical clipping.

**Figure 5:**
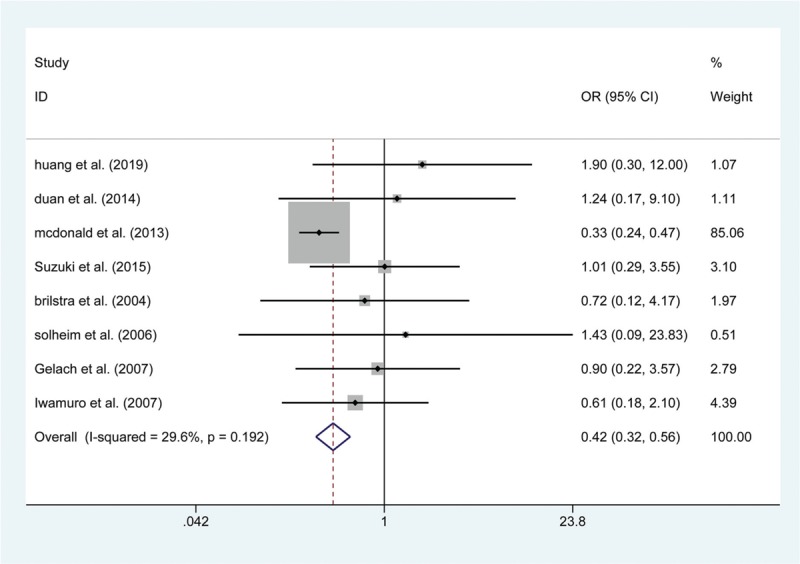
Forest plot of odds ratio (OR) of postoperative ischemia at short-term follow with endovascular coiling versus surgical clipping.

A total of 15 articles reported data on the risk of postoperative death. The results show that the risk of death within 30 days was not significantly greater in the endovascular coiling group (OR: 1.411; 95% CI (0.875, 2.276); *P* = .157, Fig. 2), and no significant difference was found in the subgroup of patients who died in hospital (RD: −0.004; 95% CI: (−0.012, 0.004); *P* = .378). Similarly, 13 articles showed a differential risk of postoperative bleeding between endovascular coiling and surgical clipping groups, but the risk of periprocedural bleeding was not significantly different between the 2 groups, either in the short-term (RD: −0.002; 95% CI (−0.005, 0.001); *P* = .133, Fig. 3) or at the 1-year follow (OR: 0.568; 95% CI (0.072, 4.459); *P* = .590). Thirteen studies assessed the short-term rates of procedural complications in 84,612 patients and found that endovascular coiling was associated with a lower incidence of complications compared with surgical clipping (4.60% versus 7.0%; OR: 0.518; 95% CI (0.433, 0.621); *P* < .001, Fig. 4), which seemed to be derived from ischemia (4.01% versus 9.08%; OR: 0.423; 95% CI (0.317, 0.564); *P* < .001, Fig. 5) rather than from vasospasm (5.32% versus 0%; RD: 0.060; 95% CI (0.000, 0.120); *P* = .048) and hydrocephalus (1.24% versus 1.38%; OR: 0.901; 95% CI (0.789, 1.030); *P* = .127) (Table 3).

**Table 3:**
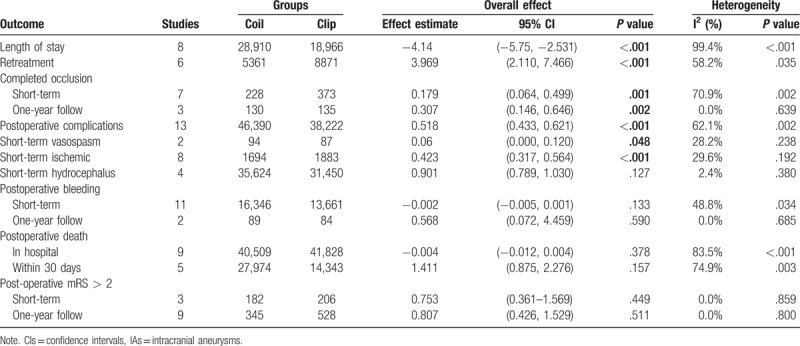
Results of meta-analysis comparison of coil and clip for the treatment of patients with unruptured IAs.

### 3.4 Secondary endpoints

**Figure 6:**
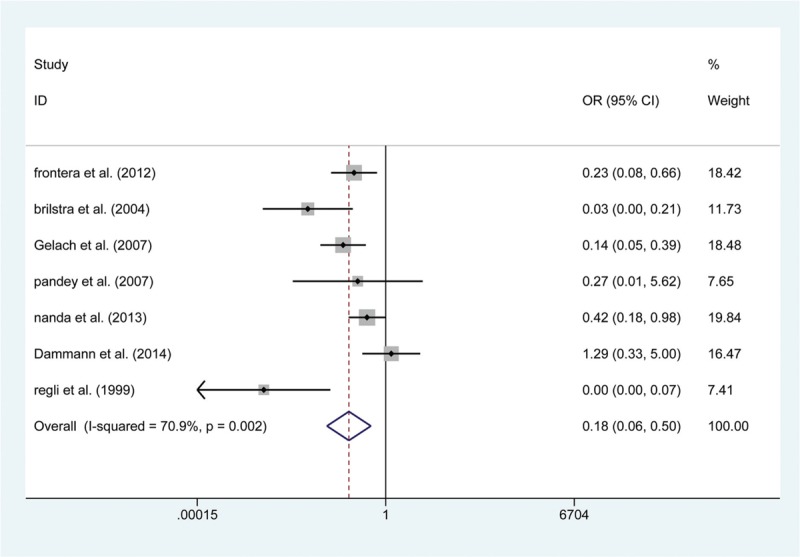
Forest plot of odds ratio (OR) of postoperative completed occlusion at short-term follow with endovascular coiling versus surgical clipping.

**Figure 7:**
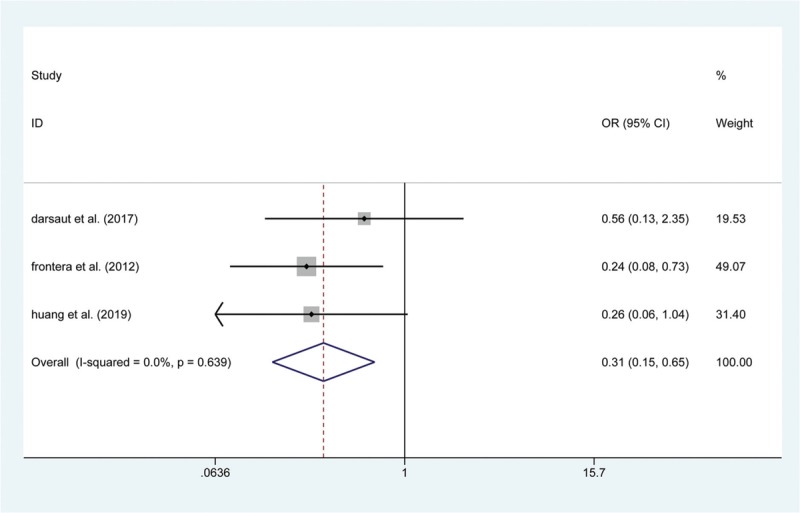
Forest plot of odds ratio (OR) of postoperative completed occlusion at 1-year follow with endovascular coiling versus surgical clipping.

The secondary endpoint, relevant to the efficiency of the procedure, was a composite of postoperative complete occlusion and mRS > 2. Similar to the results described above, 10 articles mentioned the incidence of postoperative mRS > 2, and pooling the data with a fixed-effects model revealed that there was no significant difference between the 2 groups in the rate of mRS > 2 at both short-term (OR: 0.753, 95% CI (0.361, 1.569), *P* = .449) and 1-year follow-ups (OR: 0.807, 95% CI (0.426, 1.529), *P* = .511). Furthermore, 9 studies focused on the postoperative complete occlusion rate. Pooling the results demonstrated that endovascular coiling had a lower rate of complete occlusion after surgery, in both short-term (OR: 0.179, 95% CI (0.064, 0.499), *P* = .001; Fig. 6) and 1-year follow-ups (OR: 0.307, 95% CI (0.146, 0.646), *P* = .002; Fig. 7).

### 3.5 Other outcomes of interest

**Figure 8:**
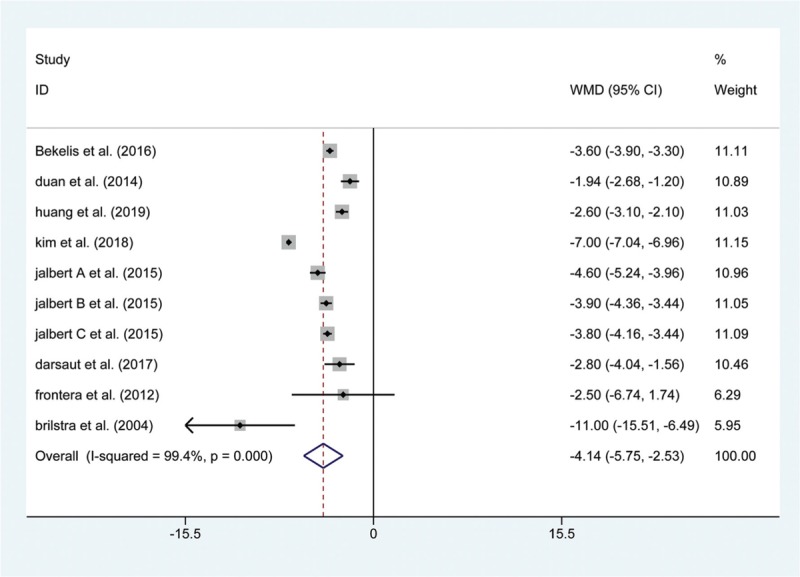
Forest plot of weighted mean difference (WMD) of the length of stay with endovascular coiling versus surgical clipping.

**Figure 9:**
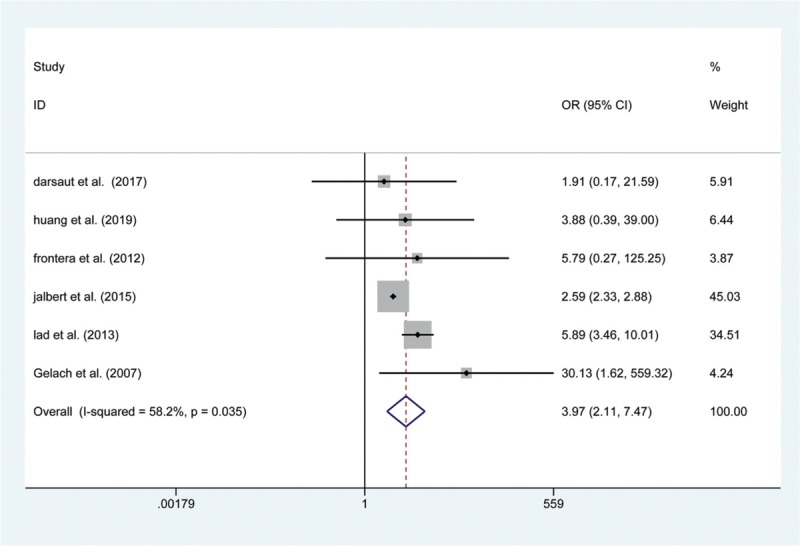
Forest plot of odds ratio (OR) of postoperative retreatment at short-term follow with endovascular coiling versus surgical clipping.

We extracted the LOS value from 8 included articles. Significant heterogeneity was observed (I^2^ = 99.4%, *P* < .001), and a random-effects model was used to show that the LOS was shorter in patients treated with endovascular coiling than in those treated with surgical clipping (WMD: −4.14, 95% CI: (−5.750, −2.531), *P* < 0.001; Fig. 8). Similarly, 6 articles mentioned the rates of retreatment; the data was pooled by a random-effects model to reveal that there was a significant difference in the rate of short-term retreatment between the 2 groups (OR: 3.969, 95% CI: (2.110, 7.466), *P* < .001; Fig. 9).

## 4 Discussion

UCAs are extremely common, with an approximate prevalence of 2% to 5%. They are increasing in their diagnosis owing to the extensive use of noninvasive imaging. Two kinds of treatment are involved in the current options to occlude UCAs. Endovascular coiling is a minimally invasive treatment in which UCA occlusion is completed through the blood vessel, while surgical clipping contained a craniotomy and clip placement on the lesion vessel to occlude UCAs. Although endovascular coiling and surgical clipping are used to prevent UCA rupture, there was no certain evidence of clinical safety and efficiency for either procedure. We conducted this meta-analysis to evaluate the clinical benefit between endovascular coiling and surgical clipping in patients with UCAs.

The primary endpoints were related to the safety in the 2 groups. Similar to the result from a previous meta-analysis,^[41]^ our results demonstrated that the pooled effect estimate of death within 30 days and death in the hospital was 1.411 (95% CI (0.875, 2.276)) and −0.004 (95% CI (−0.012, 0.004)), respectively, revealing that no significant difference was found in the incidence of mortality between the subgroup that died within 30 days and the subgroup that died in the hospital. Reassuringly, the incidence of bleeding at the short-term follow-up was similar between the 2 treatment groups; the postoperative bleeding rate was 1.65% in patients treated with endovascular coiling compared with up to 1.93% in patients treated with surgical clipping, and these bleeding rates were similar at the 1-year follow-up. Moreover, several publications,^[42,43]^ including our own, have shown endovascular coiling to be related to a lower rate of complication compared with surgical clipping (OR: 0.518; 95% CI (0.433, 0.621); *P* < .001), which seems to have derived primarily from ischemia. It has been reported that cerebral ischemia is caused by emboli escaping from aneurysms, and SAH could occur subsequently after an ischemic event.^[44,45]^ Furthermore, it has been documented that endovascular coiling was associated with a higher rate of thromboembolic events with both the coil mass and catheter as a potential thromboembolic causes,^[46]^ which is not the case with surgical clipping. Therefore, endovascular coiling may bear a higher risk of ischemia compared with surgical clipping.

The secondary endpoints referred to the effectiveness of the 2 treatments. In our study, we regarded postoperative complete occlusion and mRS > 2 as measures of surgical effectiveness. Gelach et al^[35]^ reported that the incidence of postoperative complete occlusion was 66.7% in the endovascular group (n = 39), while it was significantly higher (93.6%) in the surgical clipping group (n = 94). Brilstra et al^[34]^ found that endovascular coiling (16/33) was related to a lower complete occlusion rate than the surgical clipping group (36/37, *P* < 001). Similar to previous publications, the results of this meta-analysis illustrated that surgical clipping could increase the complete occlusion rates compared with those in the endovascular group. Meanwhile, the present meta-analysis demonstrated that surgical clipping was not associated with a higher rate of mRS > 2 than endovascular coiling for the treatment of UCAs in both short-term (OR: 0.753, 95% CI (0.361, 1.569), *P* = .449) and 1-year follow-up (OR: 0.807, 95% CI (0.426, 1.529), *P* = .511).

Although no significant difference in procedural retreatment rates was found in previous studies, different results were observed in our study (endovascular coiling 19.0% vs. surgical clipping 8.3%). This may be due to the higher rate of emboli escaping from aneurysms. Additionally, we compared the results of LOS and revealed a significantly shorter LOS in UCA patients treated by endovascular coiling than in those treated with surgical clipping, because endovascular coiling is a minimally invasive treatment without a craniotomy. However, a significant heterogeneity was observed (I^2^ = 99.4%, *P* < .001), this resulted from the different treatment strategies of different research institutions. The present study has several potential limitations. The current study only compares 10 outcomes relevant to complications of the 2 interventions due to the relatively limited data with the same long-term follow-up period. Some heterogeneity was found among the included studies, and we were unable to perform a subgroup analysis based on patient characteristics.

## 5 Conclusion

The latest evidence illustrates that surgical clipping resulted in lower retreatment rates and was associated with a higher incidence of complete occlusion, while endovascular coiling was associated with shorter LOS and a lower rate of complications, especially ischemia.

## Author contributions

**Data curation:** Wei Wei.

**Formal analysis:** Yi lei, Wei Wei.

**Investigation:** Yi lei, Wei Wei.

**Methodology:** Yi lei.

**Software:** Yi lei.
